# Assessing risk perception and knowledge gaps of tick-borne diseases in Nei Mongol Zizhiqu and Northeast China

**DOI:** 10.1016/j.soh.2026.100149

**Published:** 2026-01-17

**Authors:** Tingting Wang, Sen Li

**Affiliations:** a School of Art Design and Media, Wuhan Huaxia Institute of Technology, Wuhan 430223, Hubei, China; b School of Environmental Science and Engineering, Huazhong University of Science and Technology, Wuhan 430074, Hubei, China

**Keywords:** Tick-borne diseases, Risk perception, Knowledge-practice gap, Health education, Spatial disparities

## Abstract

**Background:**

Ticks are key vectors of zoonotic diseases in the Northern Hemisphere, including China, yet surveillance and public awareness remain limited. While global studies address risk perception, similar research in China, especially with spatial or longitudinal detail, is scarce. This study assesses tick-borne disease risk perception, influencing factors, and spatial variation in Northeast China and Nei Mongol Zizhiqu (also known as Inner Mongolia Autonomous Region) to inform targeted interventions.

**Methods:**

In 2019, a cross-sectional questionnaire surveyed 4000 adults in Heilongjiang, Jilin, Liaoning, and Nei Mongol Zizhiqu using multi-stage sampling. Knowledge was assessed in four domains: tick biology/ecology, bite treatment, tick-borne diseases, and bite prevention, alongside socio-demographic and behavioral data. Descriptive statistics and multiple logistic regression identified knowledge levels and associated factors.

**Results:**

Knowledge of tick biology was relatively high (1830/4000, 45.8% with high knowledge), but awareness of bite treatment, diseases, and especially prevention was low (31.5% with high tick-borne disease knowledge; 21.6% with high prevention knowledge), even among high-risk groups. Urban residents had higher knowledge than those in rural or remote areas. Frequent woodland visits and prior tick bites increased knowledge of tick biology (regression coefficients: 0.311 and 0.387, both *P* < 0.001) but not prevention. Education and outdoor activity showed mixed associations with knowledge domains.

**Conclusion:**

Major gaps exist in public knowledge of tick-borne diseases, particularly regarding prevention, with notable disparities across regions and risk groups. Targeted, region-specific interventions are urgently needed to improve awareness and protection, especially in high-risk and low-awareness areas.

## 1 Introduction

Ticks are key vector organisms widely distributed across temperate regions of the Northern Hemisphere, serving as carriers for numerous zoonotic diseases and posing significant challenges to public health security [1,2]. In China, ticks are found in nearly all provincial-level administrative regions (except the Macau Special Administrative Region) [3], with predominant species such as *Haemaphysalis longicornis*, *Dermacentor silvarum*, *D*. *nuttalli*, *Ixodes persulcatus*, *H*. *conicinna*, *Rhipicephalus microplus*, and *R. sanguineus* s.l., transmitting a wide spectrum of pathogens. These include *Borrelia burgdorferi s.l.* (the agents of Lyme disease, approximately 20 % prevalence in *I. persulcatus* in the northeast), tick-borne encephalitis (TBE) virus, severe fever with thrombocytopenia syndrome (SFTS) virus, *Rickettsia japonica* and other spotted fever group rickettsiae, Crimean-Congo hemorrhagic fever (CCHF) virus, *Babesia* spp., *Anaplasma* spp., and *Ehrlichia* spp. [4,5]. Risks are region-specific: Lyme disease and TBE occur mainly in forested northeast; CCHF and nairoviruses in arid northwest grasslands; spotted fever clusters in central/eastern mountains; and diverse protozoa and bacteria in the south/southwest, some without confirmed human cases [5,6]. In recent years, profound changes in land-use patterns—such as rapid urbanization and large-scale reforestation—have contributed to increasingly complex ecological dynamics of tick-borne disease transmission. Strengthening surveillance and implementing effective control measures have thus become urgent priorities in the field of public health.

Tick-borne diseases are an increasing public health concern worldwide, and understanding risk perception is essential for effective prevention. In Europe, general practitioner consultations for tick bites and Lyme borreliosis in the Netherlands rose sharply from 1994 to 2009, with the highest incidence consistently in children (particularly boys aged 5–9 years) who also showed good knowledge of these diseases and valued protective measures such as body checks [7]. In North America, the U.S. surveillance data show that tick-borne diseases more than doubled between 2004 and 2016, totaling 642,602 reported cases of 16 vector-borne diseases, with Lyme disease comprising 82 % [8]. Insurance claims suggest around 476,000 Lyme disease diagnoses and treatments annually in 2010–2018, far exceeding surveillance counts and underscoring the true burden [9]. In Asia, China reported 34,063 cases of SFTS and 4178 cases of TBE from 2005 to 2024, reflecting growing recognition and reporting of tick-borne pathogens [10]. Collectively, these regional patterns highlight that despite differences in pathogens and incidence, tick-borne diseases are a significant and expanding global challenge, making public awareness and effective risk communication critical components of prevention. Given the absence of an effective vaccine for Lyme disease, prevention strategies primarily rely on environmental management and health education to reduce human exposure to infected ticks while enhancing self-protection awareness among high-risk populations [11]. Health education, particularly guidance on personal protective behaviors, such as avoiding high-risk tick habitats, wearing protective clothing, using repellents correctly, and thoroughly checking the body after outdoor activities, is widely recognized as one of the most cost-effective approaches to preventing tick-borne diseases [12,13].

In contrast, research on risk perception of tick-borne diseases in China remains comparatively underdeveloped. A review of domestic literature from the past two decades reveals that most studies focus on the ecological characteristics of ticks and pathogen detection, while only a limited number address human risk perception [14]. Existing perception studies are predominantly descriptive cross-sectional surveys, lacking longitudinal analytical frameworks based on dynamic monitoring data, as seen in countries like the Netherlands [15] and France [16]. Regarding geographic disparities in knowledge, while certain tick-borne diseases (e.g., severe fever with thrombocytopenia syndrome, SFTS) are known to be concentrated in specific provinces [17,18], quantitative assessments of high-risk behaviors, such as outdoor activity frequency and adoption of personal protective measures, and their spatial distribution remain insufficient. A persistent disconnection exists between current tick control measures and public awareness or acceptance, as evidenced by substantial gaps in understanding and public concerns about the implementation of preventive strategies, limited support for suppression activities, and the inconsistent adoption of recommended behaviors, which is a pattern observed across multiple regions and highlighted by both empirical surveys and multi-national comparative studies [[19], [20], [21], [22], [23]]. These gaps in risk perception not only hinder the development of targeted health education strategies for high-risk populations but also complicate efforts to address emerging risks, such as habitat fragmentation of tick vectors due to urbanization.

To address these research gaps, this study focuses on the three provinces in Northeast China and the Nei Mongol Zizhiqu (also known as Inner Mongolia Autonomous Region) , an established hotspot for both established and emerging tick-borne diseases in China, to systematically assess local residents' risk perception of tick-borne diseases and explore its influencing factors. The specific objectives of this study include: (1) identifying and supplementing key dimensions of tick-borne disease risk perception that have been underexplored, based on existing disease prevention theories and tailored to China's context, and collecting empirical data through large-scale surveys; (2) conducting in-depth analyses of spatial heterogeneity in knowledge, attitudes, and behaviors related to ticks among different population groups, as well as multi-dimensional influencing factors (including socio-demographic characteristics and environmental exposure); (3) providing scientific evidence and decision-making support for public health authorities to develop more targeted and publicly acceptable tick-borne disease prevention measures and health education strategies.

## 2 Methods

### 2.1 Research area, subjects, and sampling strategy

The survey was conducted in Heilongjiang, Jilin, and Liaoning Provinces, and the Nei Mongol Zizhiqu—one of China's major endemic foci for tick-borne diseases. This high-risk area harbors key vectors such as *I. persulcatus*, *H. longicornis*, and *D. silvarum* (Zhang et al., 2019; Zhao et al., 2021), which transmit *B. burgdorferi* s.l., TBE virus, SFTS virus, and numerous emerging pathogens, including 11 newly identified viruses. The region shows high *B. burgdorferi* prevalence in ticks (about 20 %), some of China's highest TBE and SFTS case rates (Dong et al., 2024; Wu et al., 2025; Liu et al., 2025), and substantial exposure risk from forestry, farming, and livestock husbandry in extensive forest–grassland ecotones such as the Changbai Mountains, Daxing'an Mountains and Hulunbuir Grasslands, making it an ideal setting to assess public awareness and preventive behavior.

Given this epidemiological importance, we conducted a cross-sectional questionnaire survey in 2019 among permanent residents aged 18 years and above across the four provincial-level regions. To ensure broad geographic distribution and representativeness of the sample, a multi-stage sampling method was employed. First, all 103 county-level administrative units within the four provincial-level regions were selected as primary sampling units (PSUs), which included 52 banners, 17 counties, 11 county-level cities, and 23 municipal districts. A minimum of 10 valid questionnaires was collected in each unit to provide baseline reliability and guarantee that no locality was omitted, thereby avoiding over-representation from a limited number of urban centers. The total sample size of 4000 was determined to balance the goal of maximum spatial representativeness with practical constraints on data collection. Including all county-level units ensured the capture of ecological and socio-demographic diversity across the study area, which is critical for the study's aims. The specific sample size distribution across provinces was as follows: Heilongjiang Province (1280 questionnaires), Jilin Province (600 questionnaires), Liaoning Province (1000 questionnaires), and Nei Mongol Zizhiqu (1020 questionnaires).

### 2.2 Data collection

The data collection for this survey was entrusted to a professional online research service platform—Wenjuanxing (Suzhou Zhongyan Network Technology Co., Ltd., Suzhou, Jiangsu, China). This company possesses a large online sample database covering the Chinese mainland, enabling efficient distribution and collection of questionnaires. During the data collection process, strict adherence to personal information protection principles was maintained. All collected data were anonymized to ensure participant privacy. To improve response rates and minimize potential selection bias, appropriate incentives were provided to participants who completed the questionnaire. The survey was conducted entirely in Chinese.

### 2.3 Questionnaire design and core variable measurement

The primary objective of the questionnaire was to assess residents' knowledge of ticks and the diseases they transmit in the study area, thereby providing empirical support for the development and optimization of disease prevention and control strategies. The questionnaire consisted of two main parts: the first part focused on evaluating participants' knowledge about ticks, while the second part collected participants’ socio-demographic background information. The questionnaire was developed following a review of relevant studies on vector-borne disease knowledge, attitudes, and practices (Buczek et al., 2020; Shadick et al., 2016), with survey items adapted from validated instruments used in previous tick-borne disease research and modified for the Chinese context. A translated full questionnaire is provided in the Supplementary Materials.

The assessment of tick-related knowledge was structured around the following four dimensions, with each dimension generating an initial knowledge score (specific questions).

(1) Knowledge of tick biology and ecology (KB): This dimension included four questions evaluating participants' understanding of tick morphology (via silhouette identification), ecological habits, primary habitats, and active seasons. Each correct answer was awarded 1 point, with a total score of 4 points for this dimension.
(2) Knowledge of tick bite treatment (KT): This dimension consisted of four questions assessing participants' mastery of correct post-bite handling methods. Each correct identification of a key treatment method or knowledge point was awarded 1 point, with a total score of 4 points for this dimension.
(3) Knowledge of tick-borne diseases (KD): This dimension included four questions evaluating participants' awareness of common diseases transmitted by ticks. Each correct identification of a disease was awarded 1 point, with a total score of 4 points for this dimension.
(4) Knowledge of tick bite prevention (KP): This dimension consisted of six questions aimed at understanding participants' knowledge of effective measures to prevent tick bites. Each correct identification of a preventive measure was awarded 1 point, with a total score of 6 points for this dimension.

### 2.4 Data analytical steps

The data analysis process began with descriptive statistics. Socio-demographic characteristics of the sample and participants' raw scores across the four tick-related knowledge dimensions were summarized to preliminarily reveal distribution patterns and potential differences in knowledge levels among groups with varying socio-demographic backgrounds. Subsequently, to explore key factors influencing residents’ knowledge of ticks, multiple binary logistic regression models were constructed. In these models, the dependent variable was the dichotomized knowledge level: participants scoring above the mean in the KB, KT, KD, and KP dimensions were defined as having relatively high knowledge levels (coded as 1), while those scoring below or equal to the mean were defined as having relatively low knowledge levels (coded as 0). Independent variables were selected using the backward stepwise method (Likelihood Ratio test), which iteratively removed variables with weaker explanatory power to construct a concise and robust predictive model. The initial pool of potential influencing factors included gender, age, education level, occupation type, daily activity exposure risk (e.g., frequency of outdoor activities), past residential location type (urban/rural), and tick bite history within the past five years. All statistical analyses were conducted using SPSS Statistics software (version 26.0).

## 3 Results

### 3.1 Characteristics of survey participants and mastery of tick-borne disease knowledge

Based on the analysis of the 4000 valid questionnaires collected, the study participants exhibited several notable characteristics (Table 1). In terms of gender composition, there were 2084 male and 1916 female respondents, with the proportions being nearly equal. Regarding age distribution, young and middle-aged adults constituted the majority, with 1318 participants aged 18–30 years and 1891 aged 31–40 years, while other age groups, particularly those aged 61 year and above, were underrepresented. In terms of educational attainment, respondents who had completed high school (1277 individuals) or obtained a bachelor's degree (1696 individuals) accounted for the largest proportions. With respect to occupational status, technical professionals (969), manufacturing workers (903), and service industry employees (705) were the predominant occupational groups. Notably, most respondents (2237) reported having lived in rural areas within the past five years but had since relocated to urban areas, while 1440 stated they had not resided in rural areas during this period, and only 323 continued to live in rural settings.

**Table 1:** Respondents’ knowledge levels on ticks and tick-borne diseases by demographic and behavioral factors.

Question	Category	Total number of responses	Knowledge of tick biology and ecology (KB)		Knowledge of treatment of tick bites (KT)		Knowledge of tick-borne disease (KD)		Knowledge of preventive measures (KP)	
			High	Low	High	Low	High	Low	High	Low
Please select your gender.	Male	2084	972	1112	972	1112	672	1412	429	1655
	Female	1916	858	1058	858	1058	587	1329	434	1482
Please select your age category.	Under 18 years	0	0	0	0	0	0	0	0	0
	18–30 years	1318	763	555	598	720	369	949	316	1002
	31–40 years	1891	1052	839	810	1081	565	1326	383	1508
	41–50 years	602	378	224	319	283	218	384	118	484
	51–60 years	173	124	49	99	74	100	73	41	132
	61–70 years	11	6	5	4	7	7	4	4	7
	Over 70 years	5	0	5	0	5	0	5	1	4
Please select your education level.	Primary school or less	231	133	98	115	116	87	144	46	185
	Middle school	376	230	146	186	190	151	225	67	309
	Completed high school	1277	749	528	588	689	405	872	264	1013
	Bachelor's degree	1696	1152	817	895	1074	580	1389	459	1510
	Master's degree or higher	147	59	88	46	101	36	111	27	120
Please select your employment status.	Senior executives	230	102	128	78	152	49	181	41	189
	Technical professionals	969	598	371	443	526	340	629	201	768
	Administrative and support staff	553	305	248	246	307	166	387	117	436
	Service industry workers	705	405	300	309	396	205	500	148	557
	Agriculture and fisheries workers	199	110	89	89	110	68	131	48	151
	Manufacturing personnel	903	572	331	461	442	298	605	190	713
	Freelancers	276	145	131	125	151	78	198	60	216
	Students	103	44	59	42	61	18	85	29	74
	Others	62	42	20	37	25	37	25	29	33
Have you lived in a rural area in the past five years?	Yes, I still live in the countryside	323	136	323	90	233	76	247	56	267
	Yes, but now I have moved to urban to live	2237	1308	2237	1022	1215	718	1519	522	1715
	No	1440	879	1440	718	722	465	975	285	1155
How often in the past five years have you done outdoor sports?	Once a week or more	698	404	294	338	360	215	483	229	469
	1–3 times a month	1502	863	639	659	843	472	1030	340	1162
	Once or twice a quarter	1152	664	488	544	608	384	768	190	962
	Less than three times a year	450	290	160	214	236	140	310	77	373
	No outdoor exercise	198	102	96	75	123	48	150	27	171
In the past five years, from May to October each year, how long have you been active in the forest each day?	Three or more hours a day	113	53	60	39	74	28	85	18	95
	1–3 h a day	923	471	452	360	563	266	657	221	702
	Less than 1 h a day	1853	1091	762	911	942	617	1236	359	1494
	Avoid these places	1111	708	403	520	591	348	763	265	846
In the past five years, have you been bitten by a tick?	I Have not been bitten by a tick	2534	1605	929	1312	1222	885	1649	646	1888
	I Have been bitten by a tick, but only a few times (1–3 times).	567	289	278	204	363	197	370	102	465
	I Have been bitten by a tick many times (more than 3 times)	86	33	53	16	70	9	77	8	78
	I'm not sure if I have been bitten by a tick	813	396	417	298	515	168	645	107	706

Regarding lifestyle habits, the largest group of respondents engaged in outdoor activities one to three times per month (1502), followed by those participating once or twice per quarter (1152), and those engaging at least once per week (698). Concerning the duration of activity in forested areas from May to October each year, the highest proportion of respondents (1853) reported spending less than 1 h per day in these environments, while a considerable number (1111) indicated that they actively avoided such areas. In terms of tick bite history, the vast majority (2534) reported never having been bitten, 567 had experienced a small number of bites (1–3 times), and 813 were uncertain about their bite history.

Analysis of tick-related knowledge levels revealed significant differences across various knowledge dimensions. Regarding the KB dimension, respondents generally performed relatively well. For example, among men (972 high/1112 low knowledge), the 18–30 years age group (763 high/555 low), the 31–40 years age group (1052 high/839 low), and those with a bachelor's degree (1152 high/544 low), the proportion of individuals with high knowledge levels was particularly prominent. Interestingly, respondents with a master's degree or higher (59 high/88 low) and those currently residing in rural areas (136 high/187 low) demonstrated comparatively lower mastery in this domain.

The scores of KT dimension varied across subgroups, but overall, low knowledge levels predominated. For instance, both men (972 high/1112 low) and women (858 high/1058 low) exhibited this trend, although among bachelor's degree holders, those with high knowledge (895) slightly outnumbered those with low knowledge (801). Of particular note, respondents with a history of tick bites—whether bitten 1–3 times (204 high/363 low) or more than three times (16 high/70 low)—demonstrated distinctly low KT scores.

The KD dimension showed a pervasive trend of low mastery, with nearly all subgroups showing a far greater number of respondents with low knowledge compared to those with high knowledge. For example, in the 31–40 years age group, only 565 had high knowledge, while 1326 had low knowledge; among bachelor's degree holders, the numbers were 580 high vs. 1116 low. Even among those who frequently engaged in outdoor activities (at least once per week: 215 high/483 low) or spent extended periods in forested areas (1–3 h per day: 266 high/657 low), KD scores remained low. Individuals with multiple tick bite experiences (9 high/77 low) had particularly poor KD scores.

The KP dimension was the weakest dimension—a striking feature. Nearly all subgroups demonstrated extremely low mastery in this area. For example, among men, only 429 had high knowledge, compared to 1655 with low knowledge; for women, the numbers were 434 high and 1482 low; in the 31–40 years age group, 383 high and 1508 low; and among bachelor's degree holders, 459 high and 1237 low. Particularly concerning is that high-risk groups—such as those engaged in agriculture and fisheries (48 high/151 low), those frequently participating in outdoor activities (at least once per week: 229 high/469 low), those spending prolonged periods in forested areas (more than 3 h per day: 18 high/95 low), and individuals with multiple tick bite experiences (8 high/78 low)—also exhibited very low KP scores.

In summary, respondents demonstrated relatively good mastery of tick biology and ecology, but knowledge in the other three areas—especially tick bite prevention measures—was generally and severely lacking, with knowledge of tick-borne diseases also being notably low. An anomalous finding was that groups with higher exposure risk (such as those living in rural areas, frequently engaging in outdoor activities, spending extended periods in forests, or having a history of tick bites) did not exhibit correspondingly higher knowledge levels in relevant domains (particularly KT, KD, and KP), and in some cases, knowledge was even lower. For example, individuals with multiple tick bite experiences had extremely low scores in KT, KD, and KP. Although there were slight differences in knowledge mastery across gender, age, and educational attainment, the overall deficiency in KP was a common feature across all groups. These results suggest that while some segments of the population possess a basic understanding of tick biology, there exists a significant knowledge gap in critical areas such as disease awareness, bite management, and—most importantly—preventive measures, which may pose a potential hazard to public health protection.

### 3.2 Spatial variation in knowledge of tick-borne disease risk in the study area

**Fig. 1:**
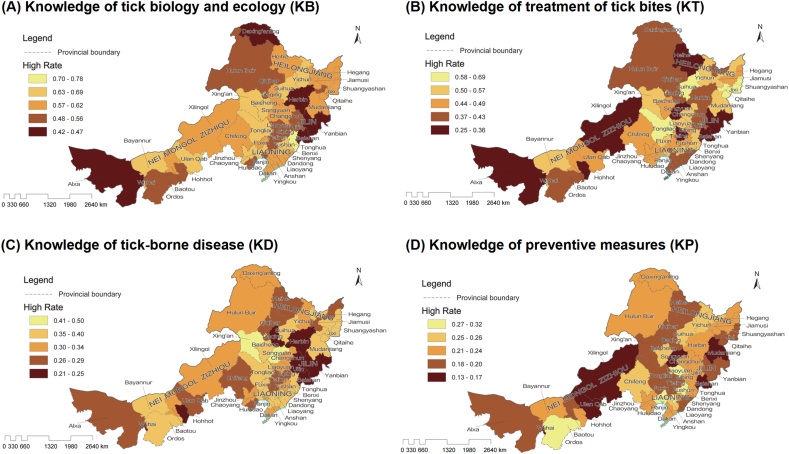
Spatial distribution of the rate of high-knowledge respondents on ticks and tick-borne diseases across surveyed regions in Northeast China and Nei Mongol Zizhiqu. A: Knowledge of tick biology and ecology (KB); B: Knowledge of treatment of tick bites (KT); C: Knowledge of tick-borne disease (KD); D: Knowledge of preventive measures (KP). Each map uses a color gradient to represent the knowledge rate of the corresponding category in different regions, where lighter colors indicate higher knowledge rates and darker colors indicate lower knowledge rates.

This questionnaire survey targeted several prefecture-level administrative regions in the three northeastern provinces of China and the Nei Mongol Zizhiqu. It systematically assessed the number of respondents in both high- and low-knowledge groups across four dimensions (KB. KT, KD, and KP). The results reveal significant distributional differences across all four knowledge dimensions, with pronounced geographic disparities in both the absolute number and the rate of high-knowledge respondents (Fig. 1).

In terms of KB, cities such as Harbin (89 high/99 low), Qiqihar (83 high/82 low), Hulunbuir (79 high/63 low), and Chifeng (78 high/47 low) had notably higher numbers in the high-knowledge group compared to other regions, indicating a strong knowledge base in these areas. Conversely, cities such as Wuhai (19 high/11 low), Alxa League (13 high/17 low), Liaoyuan (23 high/19 low), and Qitaihe (23 high/18 low) demonstrated generally lower knowledge levels. However, when considering the rate of high-knowledge respondents (Fig. 1), some smaller cities such as Tonghua and Wuhai show comparatively high KB rates despite their smaller sample sizes. For KT, Harbin (80 high/108 low), Qiqihar (67 high/98 low), and Hulunbuir (61 high/81 low) again showed a higher absolute number of respondents in the high-knowledge group, outperforming areas like Wuhai (17 high/13 low), Alxa League (9 high/21 low), and Liaoyuan (23 high/19 low). Regarding KD, Harbin (40 high/148 low), Qiqihar (44 high/121 low), Hulunbuir (43 high/99 low), and Chifeng (36 high/89 low) had substantially more respondents in the high-knowledge group than other regions, while Wuhai (7 high/23 low) and Alxa League (8 high/22 low) lagged behind. For KP, Harbin (42 high/146 low), Qiqihar (30 high/135 low), Hulunbuir (32 high/110 low), and Chifeng (30 high/95 low) again stood out, whereas Wuhai (4 high/26 low), Alxa League (6 high/24 low), Liaoyuan (9 high/33 low), and Qitaihe (8 high/33 low) had relatively low awareness in terms of both umber and rate.

Geographical analysis indicates that central cities in the three northeastern provinces—such as Harbin, Qiqihar, Changchun, Shenyang, and Dalian—consistently exhibit larger high-knowledge groups but only moderate high-knowledge rates across all four dimensions. This may be attributed to the widespread distribution of ticks, higher incidence of tick-borne diseases, and more intensive health education campaigns in these areas. Within the Nei Mongol Zizhiqu, internal disparities are evident: Hulunbuir, Chifeng, and Xilin Gol League have a higher proportion of high-knowledge respondents, while the western regions, such as Alxa League and Wuhai, display generally lower rates in KD and KP despite relatively higher rates in KB and KT, reflecting uneven regional outreach and education on prevention and control. Overall, prefecture-level cities in the eastern (northeastern) provinces demonstrate higher absolute numbers of informed respondents but not always the highest high-knowledge rates compared to some western leagues and cities, particularly Alxa League and Wuhai, which consistently show contrasting patterns across all knowledge dimensions and thus require strengthened health education and capacity building. Moreover, cities with greater economic development and population density (e.g., Harbin, Shenyang, Dalian, and Changchun) tend to have larger high-knowledge groups rather than consistently higher rates than less-developed rural areas, revealing a certain degree of urban-rural disparity.

In summary, Harbin, Qiqihar, Hulunbuir, and Chifeng maintain relatively high absolute numbers of respondents in the high-knowledge group, likely due to high tick prevalence, substantial disease burden, and heightened public attention. In contrast, Alxa League, Wuhai, Liaoyuan, and Qitaihe exhibit relatively small high-knowledge groups and low rates in KD and KP, and are in urgent need of enhanced health education. The spatial distribution of awareness across the four knowledge dimensions, as illustrated in Fig. 1, reflects both the concentration of high-knowledge respondents and marked variation in high-knowledge rates, underscoring the necessity of tailoring health education to local conditions and prioritizing outreach and capacity building in areas with lower knowledge levels.

### 3.3 Factors influencing risk knowledge

This study employed multi-variate logistic regression analysis to explore the impact of various demographic and behavioral factors on the four dimensions of tick-related knowledge (KB, KT, KD, and KP) (Table 2). The results indicated a significant positive correlation between the frequency of forest visits and KB scores (coefficient = 0.311, *P* < 0.001). Individuals with prior tick bite experiences also scored significantly higher in this knowledge dimension (coefficient = 0.387, *P* < 0.001). In terms of occupation, administrative and logistical personnel (coefficient = 0.212, *P* = 0.024) and students (coefficient = 0.732, *P* < 0.001) demonstrated better scores in knowledge of tick biology and ecology. The associations with gender (male, coefficient = −0.117, *P* = 0.070) and being over 60 years of age (coefficient = 0.989, *P* = 0.057) showed a trend toward statistical significance but did not reach the conventional threshold (*P* < 0.05).

**Table 2:** Factors influencing four dimensions of tick-related knowledge based on multi-variate logistic regression analysis.

Variable^a^	Knowledge on tick biology and ecology (KB)		Knowledge of treatment of tick bites (KT)		Knowledge of tick-borne disease (KD)		Knowledge of preventive measures (KP)	
	Coefficient	*P*	Coefficient	*P*	Coefficient	*P*	Coefficient	*P*
Gender (male)	−0.117	0.070	0.114	0.078				
Age (over 60 years)	0.989	0.057	−1.100	0.059				
Education (primary and lower)			0.159	0.077	0.395	0.000	−0.192	0.091
Outdoor activists							0.698	0.000
Frequent woodland visits	0.311	0.000					−0.437	0.000
Lived in rural areas			−0.164	0.016			0.240	0.008
Resides in Liaoning Province							0.175	0.046
Resides in Heilongjiang Province			0.130	0.057				
Had tick bites	0.387	0.000			−0.597	0.000	−0.434	0.000
Occupation (administration and supportive)	0.212	0.024						
Occupation (student)	0.732	0.000			−0.709	0.007		
Constant	−1.911	0.001	−0.101	0.131	−0.827	0.000	−1.506	0.000

a Note: Blank co-efficient values indicate these variables are not retained in the final models after backward stepwise selection.

Regarding KT, residing in rural areas was negatively correlated with knowledge scores (coefficient = −0.164, *P* = 0.016), suggesting a deficiency in this knowledge among rural residents. Individuals with an education level of elementary school or below (coefficient = 0.159, *P* = 0.077), those over 60 years old (coefficient = −1.100, *P* = 0.059), and residents of Heilongjiang Province (coefficient = 0.130, *P* = 0.057) showed trends toward statistical significance in relation to this knowledge dimension. Additionally, the association between males (coefficient = 0.114, *P* = 0.078) and knowledge of tick bite management was also close to statistical significance.

In the dimension of KD, individuals with an education level of elementary school or below scored significantly higher (coefficient = 0.395, *P* < 0.001). Conversely, having prior tick bite experiences (coefficient = −0.597, *P* < 0.001) and being a student (coefficient = −0.709, *P* = 0.007) were significantly negatively correlated with scores in this knowledge dimension.

The analysis of the KP dimension revealed that individuals who engage in outdoor activities scored significantly higher than those who do not (coefficient = 0.698, *P* < 0.001). However, frequent entry into forested areas (coefficient = −0.437, *P* < 0.001) and having prior tick bite experiences (coefficient = −0.434, *P* < 0.001) were negatively correlated with scores in this knowledge dimension. Residing in rural areas (coefficient = 0.240, *P* = 0.008) and in Liaoning Province (coefficient = 0.175, *P* = 0.046) positively correlated with knowledge of preventive measures against tick bites, while having an education level of elementary school or below (coefficient = −0.192, *P* = 0.091) approached significance in its negative correlation with this knowledge dimension.

In summary, personal behavioral experiences (such as frequent entry into forested areas and prior tick bites) and socio-demographic characteristics (such as occupation, education level, and place of residence) significantly influence levels of tick-related knowledge. The direction and degree of the effects of different factors vary across knowledge dimensions. Overall, relevant experiences and socio-demographic factors are crucial determinants of tick-related knowledge levels, highlighting the urgent need for targeted health education interventions.

## 4 Discussion

This study developed a comprehensive assessment system for tick-related knowledge, including biology and ecology, bite management, disease awareness, and preventive measures. This approach addresses the limitations of previous studies that focused on only one aspect. Logistic regression revealed significant differences in the factors influencing each knowledge area. KP scores were particularly low and inversely associated with high-risk behaviors, emphasizing the need for targeted health education. By surveying 103 county-level units in Northeast China and Nei Mongol Zizhiqu, the study identified clear spatial differences in knowledge levels. When evaluated by the rate of high-knowledge respondents (rather than absolute numbers), residents in eastern central cities demonstrated higher awareness compared to those in remote western regions. Based on these findings, the study recommends prioritizing interventions in areas with lower knowledge levels. Notably, high-risk groups exhibited lower knowledge of preventive measures, which challenges the assumption that experience leads to better understanding and provides new support for behavioral intervention strategies. Overall, while residents showed reasonable knowledge of tick biology and ecology, there were widespread gaps in bite management, disease awareness, and preventive measures. High-risk individuals, such as rural residents and frequent participants in outdoor activities, did not outperform low-risk groups in these areas and sometimes had even lower knowledge. This persistent knowledge gap in prevention and control may pose a significant threat to regional public health.

Our analysis shows that male respondents, those aged 18–40 years, and those with a bachelor's degree had higher KB scores, while rural residents and those with a master's degree or above scored lower. Previous studies similarly report that education level and lifestyle influence tick biology knowledge; for example, rural residents often have limited understanding of tick life cycles, shaped by habitual exposure [24,25]. Lower KB scores among rural residents may reflect habitual exposure that normalizes ticks as everyday pests rather than disease vectors, leading to reliance on local experience over formal health information [26]. This issue is potentially exacerbated by limited access to targeted health campaigns in rural areas [27]. Lower scores among those with master's degrees may be linked to lifestyle, as many work in urban, office-based, or academic settings with minimal contact with tick habitats [28]. This contrasts with bachelor's degree holders, who are more frequently employed in outdoor or field-based occupations that involve consistent exposure to ticks. A significant gender difference was observed, with men scoring higher, which is an uncommon finding in the literature [29]. This finding likely reflects regional occupational patterns, as men are often more engaged in fields such as agriculture, forestry, animal husbandry, or construction, which entail regular exposure to tick-prone environments [[30], [31], [32]]. Age showed a similar influence: participants aged 18–40 years scored higher than older adults, possibly due to greater digital literacy, more access to online health resources, higher levels of outdoor activity, and more recent exposure to health messaging [33].

KT scores were generally low, especially among individuals with a history of tick bites, indicating limited practical response skills. Other research also highlights inadequate knowledge and misconceptions about tick bite treatment in rural populations, including among parents of exposed children [24,25]. KD scores were low across all subgroups, with few individuals showing high awareness. This highlights an urgent need to improve public understanding of tick-borne diseases. Inadequate knowledge is common across regions, regardless of endemic status, and recognition of diseases like Lyme disease and TBE remains incomplete, influenced by age and previous tick exposures [26,29,34]. The lowest scores were in KP dimension, even among high-risk groups such as agricultural and fishery workers, forest activity participants, and those with prior tick bites. This reflects a major gap between protective knowledge and actual needs. Field studies confirm poor adoption of preventive behaviors among those most at risk, due to perceived inconvenience, low risk perception, and insufficient training or resources. For example, forest workers, farmers, and outdoor laborers often underutilize protective clothing and repellents despite frequent exposure [35,36]. These findings together suggest that public health education in China should be tailored not only by geographic risk but also by demographic factors, with particular focus on older adults, urban postgraduate-educated populations, and rural communities with high exposure but low formal knowledge.

This study reveals significant behavioral heterogeneity in the structure of tick-related knowledge. Although high-risk groups demonstrated higher scores in KB dimension (coefficient = 0.311, *P* < 0.001; coefficient = 0.387, *P* < 0.001), their scores for KP dimension were significantly lower than those of low-risk groups (coefficient = −0.437 to −0.434, *P* < 0.001). This pattern is consistent with findings from other studies, which report that individuals in high-risk groups or those with prior tick exposure often possess greater biological knowledge but have a poorer understanding of, and engagement with, preventive measures. For instance, both medical students and the general population have exhibited this gap, suggesting systemic challenges in translating knowledge into protective behaviors [21,37]. This cognitive-behavioral discrepancy closely aligns with the “knowledge-attitude-practice gap” described in the Health Belief Model [38], indicating that the dissemination of knowledge alone is insufficient to effectively promote protective behaviors. For example, students achieved high scores in the KB dimension (coefficient = 0.732, *P* < 0.001), but lacked practical knowledge in areas such as tick bite management and tick-borne diseases, reflecting an education model that emphasizes theoretical mastery over applied skills. In the Chinese context, formal curricula in both school and professional training place strong emphasis on academic knowledge—often within broader biology or infectious disease modules—while practical prevention behaviors, such as personal protective measures for vector exposure, receive less direct coverage. Similarly, public health communication strategies in China have traditionally prioritized broad disease prevention messaging rather than tailored, behavior-specific training for particular at-risk groups. This focus may help explain why even well-informed individuals demonstrate low adoption of preventive practices.

This study is the first to identify the geographical gradient in tick-related knowledge levels across Northeast China and Nei Mongol Zizhiqu. Many of the comparative studies we previously cited [[24], [25], [26],^29^,^34^], were conducted outside China and mainly in Europe and South America, underscoring both the scarcity of relevant research in the Chinese context and the novelty of our findings. However, our spatial analysis also shows that cities with larger populations (e.g., provincial capitals) do not always have the highest rates of high-knowledge respondents, even though they often have the largest absolute numbers. The proportion of individuals with high levels of knowledge was significantly greater in provincial capital cities (e.g., Harbin and Shenyang) and economically developed eastern regions (47 %–55 % in KB and KT) compared to remote western areas such as Alxa League (30 %–43% depending on the dimension) and Wuhai City (13 %–63% with particularly low rates in KD and KP). Several factors may contribute to this spatial disparity. First, differences in disease burden play a role; for example, the annual incidence of TBE in Heilongjiang is higher than that in western Nei Mongol Zizhiqu [39]. Second, public health resources are unevenly distributed, with a higher density of Chinese Center for Disease Control and Prevention personnel in the three northeastern provinces than in western Nei Mongol Zizhiqu [40]. Third, the efficiency of information dissemination varies, as new media health education coverage is more extensive in provincial capitals than in pastoral areas [41,42]. Comparable patterns of geographic heterogeneity in tick knowledge and preventive behaviors have been observed elsewhere, with urban or more developed areas generally exhibiting greater awareness than rural or remote regions. Studies from the United States and Europe indicate that urban-to-rural gradients influence perceived susceptibility, knowledge, and the adoption of preventive measures, likely due to differences in access to information and local disease risk [24,43]. Given these disparities, it is recommended to prioritize the development of integrated “online + offline” education networks in regions with low knowledge levels and to enhance community emergency response training in high-incidence areas for more targeted interventions [44]. Strengthening community-level emergency response capacity aligns with recognized needs for improving practical tick control behaviors and effectively reducing disease risk [45].

The limitations of our studies are as follows. First, although the total sample of 4000 may seem modest for the study area, it was intentionally spread across all 103 county-level units to maximize representativeness. This broad rural–urban coverage supports our regional-level focus but may produce wider confidence intervals for some subgroups; larger future samples could enable finer spatial analysis. Second, individuals aged 61 and above accounted for only 0.4 % of the sample, and permanent rural residents were underrepresented (8.1 %), which may affect the representativeness of the findings for key populations. Third, the knowledge assessment tool has not yet reported reliability and validity indicators, and the cross-sectional design limits causal inference; for example, the relationship between students’ knowledge levels and educational background requires further verification through longitudinal research. Additionally, reliance on online platforms may have resulted in insufficient sampling from remote areas, and self-reported tick bite history is subject to recall bias (20.3 % of respondents were unsure whether they had been bitten), potentially affecting the accuracy of analyses relating risk exposure to knowledge levels. Future work should increase high-risk population coverage, validate assessment tools, and use mixed-methods approaches to strengthen rigor and generalizability. Nevertheless, as one of the first studies of its kind in China, this work provides a baseline for public knowledge, attitudes, and practices on tick-borne diseases, laying essential groundwork for future research and targeted prevention efforts.

## 5 Conclusion

This study highlights a significant knowledge gap among residents in Northeast China and Nei Mongol Zizhiqu regarding tick-borne diseases, particularly in areas such as bite management, disease awareness, and preventive measures. While high-risk groups (e.g., rural residents and frequent outdoor participants) demonstrated familiarity with tick ecology, their adoption of preventive behaviors remains inadequate, revealing a critical disconnect between knowledge and action. Spatial analysis further reveals disparities, with knowledge levels in western pastoral regions lagging behind those in eastern urban centers by dimension‑specific margins that are especially pronounced for decision-making and practice-related knowledge, reflecting uneven public health resource distribution. To address these gaps, targeted interventions—such as practical training for occupational risk groups and culturally tailored health education programs—are urgently needed. Despite limitations in sample representation and self-reported data reliability, these findings provide essential evidence for refining precision prevention strategies in tick-endemic regions globally. Future research should incorporate longitudinal designs and serological validation to strengthen the link between knowledge, behavior, and disease outcomes.

## CRediT authorship contribution statement

**Tingting Wang:** Writing – original draft, Visualization, Validation, Software, Methodology, Investigation, Formal analysis, Data curation, Conceptualization. **Sen Li:** Writing – review & editing, Visualization, Validation, Supervision, Resources, Project administration, Investigation, Funding acquisition, Conceptualization.

## Ethical considerations

This study is an anonymous online survey and does not contain any data which can identify individual. All participants provided informed consent before participating in the study and could choose to terminate their participation in the study at any time. The Ethical Statement was attached when the article was submitted.

## Funding

This study was supported by the 10.13039/501100001809National Natural Science Foundation of China (grant numbers 42477465 and 42107458) and 10.13039/501100003819Hubei Provincial Natural Science Foundation of China (grant number 2024AFB591).

## Declaration of competing interest

The authors declare that they have no known competing financial interests or personal relationships that could have appeared to influence the work reported in this paper.
