# Supplementary material for: Assessing risk perception and knowledge gaps of tick-borne diseases in Nei Mongol Zizhiqu and Northeast China
Source: Sci One Health. 2026 Jan 17;5:100149. doi: 10.1016/j.soh.2026.100149 (PMC12919280; doi:10.1016/j.soh.2026.100149)
Supplement: Multimedia component 1 [file mmc1.docx]

**Survey on Awareness of Ticks and Tick-Borne Diseases**

**Survey Instructions:**

The purpose of this survey is to understand the awareness level of residents in certain regions of China regarding ticks and tick-borne diseases. The research results will support decision-making for tick-borne disease prevention and control.

We appreciate your support for scientific research and your careful completion of the questionnaire.

Your personal information will remain confidential.

**Questions:**

**Section 1: About Ticks**

1.1 From which sources have you heard about ticks? [Multiple Choice]

A. Relatives or friends

B. Internet, TV, newspapers, magazines, etc.

C. Healthcare professionals

D. School education

E. Other

F. Never heard of ticks

1.2 Which of the following silhouettes represent ticks? [Multiple Choice]

A.
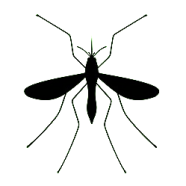


B.
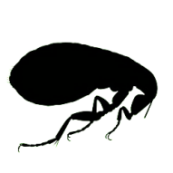


C.
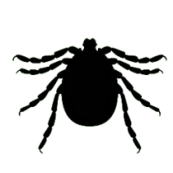


D.
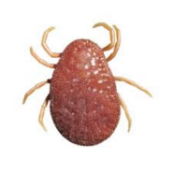


E. I don’t know

1.3 Which of the following statements about ticks are correct? [Multiple Choice]

A. Ticks can fly

B. Ticks can jump high

C. Ticks feed on blood

D. Ticks bite humans

E. Ticks bite various animals, including rodents and birds

F. Ticks can be as small as sesame seeds

G. Ticks can be as large as red beans

H. I don’t know

1.4 Where do you think ticks are most likely encountered? [Single Choice]

A. Forests and fields

B. Urban squares

C. Floors and beds at home

D. I don’t know

1.5 During which season are ticks most active? [Single Choice]

A. May to October

B. November to April

C. I don’t know

**Section 2: Experience and Risk Perception of Tick-Borne Diseases**

2.1 In the past 5 years, have you been bitten by a tick? [Single Choice/Fill in the blank]

A. No

B. Yes, but rarely (1–3 times). If remembered: How many times? _____. On average, how many ticks were found biting each time? _____

C. Yes, frequently (more than 3 times). If remembered: How many times? _____. On average, how many ticks were found biting each time? _____

D. Unsure

2.2 In the past 5 years, did you get sick after a tick bite? [Single Choice/Fill in the blank]

A. No illness after a bite

B. Fell ill but did not seek medical care

C. Fell ill and was diagnosed by a doctor as: [Fill in the blank] __________

2.3 Have people or animals around you been bitten by ticks? [Multiple Choice]

A. Family members living with me

B. Relatives not living with me

C. Colleagues

D. Friends (excluding colleagues)

E. Livestock I raise

F. Pets I raise

G. None of the above

H. I don’t know

2.4 What should be done after a tick bite? [Multiple Choice]

A. Wait for the tick to detach naturally

B. Rinse with plenty of water

C. Remove the tick entirely with specialized tools

D. Seek medical assistance

E. I don’t know

2.5 Which diseases can result from tick bites? [Multiple Choice]

A. Malaria

B. Lyme disease

C. Tick-borne encephalitis

D. Avian flu

E. Q fever

F. Dengue fever

G. I don’t know

2.6 What can be done to prevent tick bites? [Multiple Choice]

A. Wear long sleeves and pants tucked into socks during outdoor activities

B. Wear light-colored clothing and check for ticks regularly

C. Stay on trails in heavily wooded areas

D. Wash skin with soap and water after returning indoors

E. Apply insect repellent to clothing

F. Consult a veterinarian for pet tick prevention

**Section 3: About You**

3.1 Gender: [Single Choice]

A. Male

B. Female

3.2 Age: [Single Choice]

A. Under 18

B. 18–30

C. 31–40

D. 41–50

E. 51–60

F. 61–70

G. Over 70

3.3 Education Level: [Single Choice]

A. Primary school or below

B. Junior high school

C. Senior high school

D. University

E. Postgraduate or above

3.4 Occupation: [Single Choice]

A. Leaders of Party organs, state agencies, mass organizations, social organizations, enterprises, and public institutions

B. Professionals (science, engineering, healthcare, education, management, social sciences, etc.)

C. Clerical and administrative staff (public security, firefighting, civil servants, etc.)

D. Service workers (transportation, hospitality, culture, etc.)

E. Agriculture, forestry, animal husbandry, fisheries workers

F. Manufacturing workers (food processing, industrial production, etc.)

G. Freelancer

H. Student

I. Other: __________

3.5 In the past 5 years, have you lived in rural areas? [Single Choice]

A. Yes, currently living in a rural area

B. Yes, but now living in an urban area

C. No

3.6 In the past 5 years, how often did you engage in outdoor activities (e.g., hiking, camping, gardening, dog walking, cycling, jogging)? [Single Choice]

A. Weekly or more

B. 1–3 times per month

C. 1–2 times per quarter

D. Less than 3 times per year

E. No outdoor activities

3.7 From May to October in the past 5 years, how much time did you spend daily in wooded areas? [Single Choice]

A. 3 hours or more

B. 1–3 hours

C. Less than 1 hour

D. Did not visit such areas

3.8 Address (fill in to neighborhood level; no specific house numbers): [Fill in the blank]

City: __________ District: __________ Road: __________ Neighborhood: __________ Years lived here: __________
