# Supplementary material for: Assessing risk perception and knowledge gaps of tick-borne diseases in Nei Mongol Zizhiqu and Northeast China
Source: Sci One Health. 2026 Jan 17;5:100149. doi: 10.1016/j.soh.2026.100149 (PMC12919280; doi:10.1016/j.soh.2026.100149)
Supplement: Multimedia component 2 [file mmc2.pdf]

研究伦理审核

项目名称: 蜱虫传染病高发地区居民对蜱虫风险认知的问卷研究（20190916）

参与标准：本研究针对中国东北部（黑龙江省、吉林省以及辽宁省）及内蒙古自治区居民开展调研。受访者需为该区域的常住居民方可参与此项研究。未满 18 岁的参与者需在家长陪同下参与此项调研。

自愿与知情同意：在问卷开始前参与者被告知此次研究的目的，并获取知情同意。在问卷开始后参与者随时可以选择终止此项研究。

隐私与数据保护：为了解中国蜱虫传染病高发地区居民对蜱虫风险认知及不同群体的蜱暴露风险差异，研究问卷将收集被试的性别、年龄、收入、健康情况等个人信息，但均将进行去标识化处理，无法识别到个人。本研究不收集参与者的姓名、地址等标识化个人信息。本研究通过收集参与者设备记录防止参与者重复访问页面重复作答，收集的设备信息因为设计隐私仅供问卷公司内部使用，不会作为任何形式的商业用途。参与者数据收集均为匿名化处理，数据存储符合规定，保障用户数据安全。研究结束后参与者数据将会被清除。

利益冲突：本研究系华中科技大学李森老师课题组调查项目，研究内容和结果不存在利益冲突。

本研究充分考虑了安全性和公平性原则，研究内容不构成对被试的伤害和风险，被试的招募安全且基于自愿和知情同意原则，并尽最大限度保护被试的隐私，研究内容和结果不存在利益冲突。

华中科技大学李森课题组

众言科技股份有限公司

2019 年 9 月 16 日

Ethical Statement

Project: A questionnaire study on the perception of tick risks among residents living in an area with a high prevalence of tick-borne diseases. (20190916)

Participation criteria: This study was conducted among residents of Heilongjiang, Jilin, and Liaoning Provinces in northeastern China. Participation in the study was limited to permanent residents of the region. Users under the age of 18 must participate with parental accompaniment.

Voluntary and informed consent: Before the start of the questionnaire, participants are informed of the purpose of this study and give their informed consent. After the questionnaire starts, participants can choose to terminate their participation in the study at any time.

Privacy and data protection: To understand the basic structure of internet health information users and the differences in online health literacy among different groups, the research questionnaire will collect personal information of the subjects such as gender, age, income, and health conditions, but all will be de-identified, making it impossible to identify individuals. This study does not collect identifiable personal information such as participants' names and addresses. In the process of questionnaire collection, this study prevents participants from visiting the page and answering repeatedly by collecting device records such as participants' cookies/IP addresses, and the collected device information is only used internally by the questionnaire company due to privacy design, not for any form of commercial purposes. According to the Personal Information Protection Law of the People's Republic of China, participant data collection is processed anonymously, data storage complies with regulations, ensuring the security of user data. After the study, participant data will be erased after three months from the survey company's side.

Conflict of Interests: There's no conflict of interests of this study.

This study fully considers the principles of safety and fairness. The research content does not constitute harm or risk to the subjects, the recruitment of subjects is safe and based on the principle of voluntary and informed consent, and it protects the subjects' privacy to the greatest extent. There are no conflicts of interest in the research content and results.

Li Sen Research Group of Huazhong University of Science and Technology

Zhongyan Technology Co., Ltd.

September 16, 2019
